# In an endotoxaemic model, antibiotic clearance can be affected by different central venous catheter positions, during renal replacement therapy

**DOI:** 10.1186/s40635-023-00516-4

**Published:** 2023-06-09

**Authors:** Anna Bandert, Miklós Lipcsey, Robert Frithiof, Anders Larsson, David Smekal

**Affiliations:** 1 Department of Surgical Sciences, Anaesthesiology and Intensive Care, Uppsala University, Uppsala, Sweden; 2 Centre for Research and Development, Uppsala University/Region Gävleborg, Gävle, Sweden; 3 Department of Anaesthesiology and Intensive Care, Gävle Hospital, Lasarettvägen 1, 80324 Gävle, Sweden; 4 Department of Surgical Sciences, Hedenstierna Laboratory, Uppsala University, Uppsala, Sweden; 5 Department of Medical Sciences, Clinical Chemistry, Uppsala University, Uppsala, Sweden

**Keywords:** Central venous catheter, Intensive care, Continuous renal replacement therapy antibiotic concentration, Sepsis, Dialysis, Acute kidney injury

## Abstract

**Background:**

In intensive care, different central venous catheters (CVC) are often used for infusion of drugs. If a patient is treated with continuous renal replacement therapy (CRRT) a second catheter, a central venous dialysis catheter (CVDC), is needed. Placing the catheters close together might pose a risk that a drug infused in a CVC could be directly aspirated into a CRRT machine and cleared from the blood without giving the effect intended. The purpose of this study was to elucidate if drug clearance is affected by different catheter placement, during CRRT. In this endotoxaemic animal model, an infusion of antibiotics was administered in a CVC placed in the external jugular vein (EJV). Antibiotic clearance was compared, whether CRRT was through a CVDC placed in the same EJV, or in a femoral vein (FV). To reach a target mean arterial pressure (MAP), noradrenaline was infused through the CVC and the dose was compared between the CDVDs.

**Results:**

The main finding in this study was that clearance of antibiotics was higher when both catheter tips were in the EJV, close together, compared to in different vessels, during CRRT. The clearance of gentamicin was 21.0 ± 7.3 vs 15.5 ± 4.2 mL/min (*p* 0.006) and vancomycin 19.3 ± 4.9 vs 15.8 ± 7.1 mL/min (*p* 0.021). The noradrenaline dose to maintain a target MAP also showed greater variance with both catheters in the EJV, compared to when catheters were placed in different vessels.

**Conclusion:**

The results in this study indicate that close placement of central venous catheter tips could lead to unreliable drug concentration, due to direct aspiration, during CRRT**.**

**Supplementary Information:**

The online version contains supplementary material available at 10.1186/s40635-023-00516-4.

## Background

Acute kidney injury (AKI) occurs in 10–60% of critically ill patients, most often in relation to sepsis [1, 2]. Of septic AKI patients, 15–20% are treated with renal replacement therapy, commonly provided in its continuous form (CRRT), in the intensive care unit (ICU) [1–4].

Central venous catheters (CVC) are routinely used in critically ill patients treated in intensive care, and, if renal replacement therapy is required, a central venous dialysis catheter (CVDC) is inserted. The CVC is used for infusion of essential drugs, such as antibiotics and vasopressors, while the CVDC enables aspiration of blood into the CRRT circuit. The tips of the two central lines are often placed in the same caval vein, in the proximal third of the superior vena cava, the right atrium or in the inferior vena cava. If the tips of the two catheters are close, there is a risk of direct aspiration of drugs into the CRRT circuit leading to a lower blood concentration of the drugs than intended [5–8]. The possibility of direct aspiration of drugs would resemble recirculation, a phenomenon mostly described in extra corporeal membrane oxygenation (ECMO) and intermittent haemodialysis, for patients with chronic kidney failure [9–12]. Whereas recirculation in ECMO leads to lower oxygenation, and in renal replacement therapy to a lower clearance of drugs and metabolites, direct aspiration will instead result in a higher clearance of drugs [9–12]. There are case reports where direct aspiration from a CVC to a CVDC during CRRT has been suspected of producing major effects on the patients [5, 6]. Kam et al. also demonstrated the possibility of direct aspiration from a CVC to a CVDC in vitro [7]. In a previous animal model, our group has shown that the removal of noradrenaline and gentamicin was substantially increased if these drugs were infused in a CVC in close proximity to the CVDC, during CRRT [8].

Sepsis-induced AKI is common in the ICU and associated with high mortality, while administering the right amount of antibiotics in sepsis is associated with better outcomes [13, 14]. Since sepsis can affect the patient´s cardiac output and distribution of intravascular blood it could, in theory, affect the risk of direct aspiration [15]. Higher cardiac output could reduce the risk, by making the blood flow faster and thereby transport the drugs infused in the CVC away from the CVDC, before they are aspirated. If, however, the patient is hypovolemic, a smaller vena cava diameter could potentially decrease the distance between the tips and consequently increase the direct aspiration. Our study was conducted to investigate whether drug clearance is affected by CVC site in relation to CVDC site, during CRRT, in an endotoxaemic model. Our hypothesis was that infusion of gentamicin and vancomycin close to the tip of the CVDC would result in higher clearance, higher dialysate concentration and a lower serum concentration, than if infusion and CVDC were in different blood vessels. We also hypothesized that, in the same setting, infusion of noradrenaline close to the tip of the CVDC would require a higher infused dose to reach a target mean arterial blood pressure (MAP) than if infusion and CVDC were in different vessels.

## Methods

### Design

A randomised, cross-over study in an experimental, endotoxaemic animal model. Primary end point was dialysate clearance of antibiotics. Secondary end points were noradrenaline dose and serum concentration of antibiotics.

### Animals

The study was performed in eighteen, 8–10-week-old piglets of the Norwegian Landrace breed. Since information about direct aspiration is scarce, the sample size was kept small without calculation of power. The experiments were performed at the Hedenstierna Laboratory (Uppsala University, Sweden), in accordance with the ARRIVE 2.0 guidelines and the guidelines of the Swedish Board of Agriculture and the European Convention on Animal Care [16]. Ethical approval was given by the Animal Ethics Board in Uppsala, Sweden (C 155/14).

### Anaesthesia and preparation

Anaesthesia was induced by injecting a mixture of tiletamine–zolazepam 6 mg/kg and xylazin 2.2 mg/kg intramuscularly, and was maintained by an infusion of sodium pentobarbital 8 mg x kg^−1^ × h^−1^ and morphine 0.26 mg /kg/h dissolved in 2.5% glucose in a peripheral vein. Rocuronium bromide 1–3 mg/kg/h was given as continuous infusion. The dose of rocuronium enabled the piglets to make small movements or trigger in the ventilator and if signs of insufficient anaesthesia occurred the anaesthesia was deepened immediately. Ringer’s acetate solution was administered at 1 ml/kg/h, resulting in a total fluid administration rate of 20–35 mL/kg/h.

A tracheotomy was performed, and the animals were mechanically ventilated (Servo-I, Maquet, Stockholm, Sweden) with an initial setting of respiratory rate 25/min, PEEP 5 cmH2O, inspired oxygen fraction 0.3, with the tidal volume that yielded a PaCO_2_ of 4.5 to 5.5 kPa at baseline. Respiratory rate was adjusted to keep PaCO_2_ of 4.5 to 6.0 kPa during the experiment. Inspired fraction of oxygen was adjusted to keep saturation above 93% measured by pulse oximetry.

A cervical artery was catheterized for pressure monitoring and blood sampling.

**Fig. 1:**
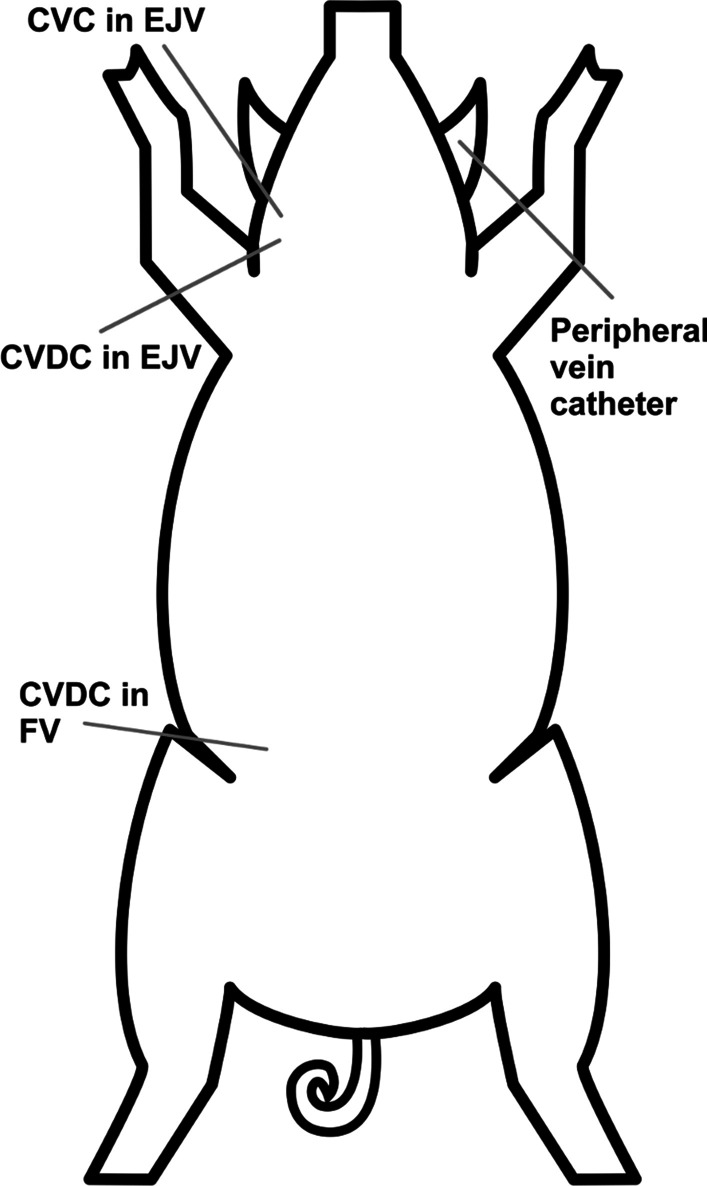
Cannulation of the animal. *CVC* central venous catheter, *CVDC* central venous dialysis catheter, *EJV* external jugular vein, *FV* femoral vein

A central venous line and a 13.5 Fr dialysis catheter were inserted through the right external jugular vein (EJV) into the SCV. An additional 13.5 Fr dialysis catheter was inserted through the femoral vein (FV) into the inferior caval vein (see Fig. 1). Catheter placement was radiographically controlled. A 7 Fr Swan–Ganz catheter was inserted in the pulmonary artery from the left EJV. A urinary catheter was introduced via a bladder incision.

### Dialysis

The CRRT machine (multiFiltrate, Fresenius Medical, Stockholm, Sweden) was prepared with a priming kit (multiFiltrate Ci-Ca® CVVHD 1000) with integrated filter (Ultraflux® AV 1000S) with a 1.8m^2^ surface area.

CRRT was started with CVVHD settings and kept throughout the experiment: blood flow 60 ml/min, dialysate flow 1200 ml/h, citrate target 4 mmol/L, post-filter ionized calcium level 0.25–0.34 mmol/L ultrafiltration 0 ml/min.

### Protocol

The pigs were anaesthetized, and prepared as stated above. At the beginning of the preparation, vancomycin (4.55 mg/ml) was started at 9 mg/kg/h for 30 min and thereafter reduced to 3 mg/kg/h throughout the rest of the experiment. Gentamicin (10 mg/ml) was infused with 3 mg/kg for 30 min and thereafter reduced to 1.5 mg/kg/h. This was calculated to give a sufficient dose to reach steady state. The infusion was initially in a peripheral vein, but was moved to the CVC in the EJV as soon as the CVC was inserted. The piglets were then randomized in pairs to start CRRT in one of the CVDC, i.e. in the EJV or FV. CRRT was started followed by a 30-min stabilization period.

After stabilization, lipopolysaccharide (LPS) was infused peripherally in a dose of 0.5 µg/kg/h. In order to let the animals react to the LPS, there was an expectation period of 60 min. An inter-individual response to LPS was expected and during this 60-min period noradrenaline (20 µg/ml) was given in the CVC, if MAP was below 60 mmHg and thereafter kept at that MAP level. Sixty minutes after start of LPS administration, the target MAP was increased to 75 mmHg for 30 min. In the piglets that already had an infusion of noradrenaline, the drug infusion was increased and in the others it was initiated, to reach and keep the target MAP of 75 mmHg.

**Fig. 2:**
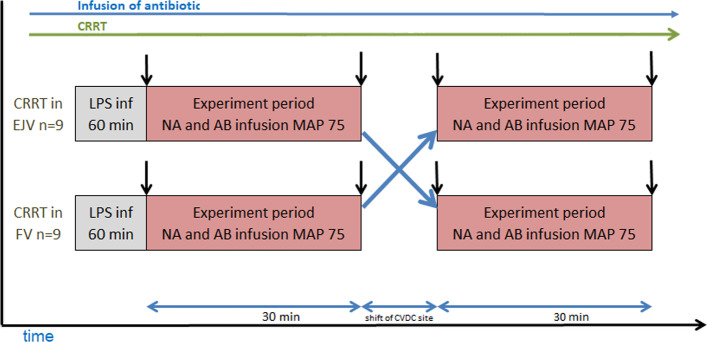
Flowchart of the method. Eighteen [18] piglets were randomized in pairs (2 × n = 9) to start continuous renal replacement therapy (CRRT) in either the external jugular vein (EJV) or the femoral vein (FV). Lipopolysaccharide (LPS) was given in a peripheral vein throughout the whole experiment in order to induce endotoxaemia. An infusion of antibiotic (AB) (Gentamicin 4.55 mg/ml and vancomycin 10 mg/ml) were started before CRRT in the EJV and kept throughout the whole experiment. An infusion of noradrenaline (NA) 20 µg/ml was started in the EJV if the mean arterial pressure (MAP) was below 60 mmHg. During the 30-min experiment period, target MAP was 75 mmHg. After 30 min, the CRRT was shifted to the other CVDC and the experiment was repeated. Black arrows indicate sampling times for dialysate and serum laboratory tests and time for registering the noradrenaline dose. In one experiment period, infusion of antibiotics and noradrenaline was close to the central venous dialysis catheter and the CRRT (i.e. in the EJV) and in the other experiment period they were in different vessels (i.e. in the FV). The animals served as their own controls

The serum and dialysate concentrations of gentamicin and vancomycin were measured before and after the 30-min period, and the administered noradrenaline dose was registered. After 30 min, the circuit was shifted to the other CVDC in the EJV or FV. The experiment was repeated, hence the piglets acted as their own controls in a cross-over design (see Fig. 2). After end of the second 30-min experiment period, pain stimuli was performed, with a pinch of a surgical forceps on the piglets hoof. If indication of insufficient anaesthesia, observed by movements, tachycardia or hypertension, the depth if anaesthesia was increased. All animals were in deep anaesthesia when euthanized by the administration of potassium chloride.

### Laboratory analyses

Serum and dialysate were obtained for analysis of gentamicin and vancomycin concentration at the start and end of the 30-min experiment period. Blood for blood gases was obtained from the arterial line, at the start of CRRT and at both the beginning and end of each 30-min experiment period. Blood gas and point care analyses were performed on an ABL 800 blood gas analyzer (Radiometer, Brønshøj, Denmark). Gentamicin and vancomycin were analysed on an Architect ci16200 (Abbott Laboratories, Abbott Park, IL, USA) with reagents (1P31) from the same manufacturer. Dialysate clearance was calculated using the formula Clearance = (dialysate concentration x dialysate flow)/ serum concentration.

### Statistical analysis methods

Data were analysed using Jamovi, Version 1.6.15.0 and Statistica Version 14. Tests for normal distribution were performed using the Shapiro–Wilk test. Normally distributed data were analysed with a paired Student’s t-test. Non-normally distributed variables were analysed using Wilcoxon signed-rank test. Testing for variance was analysed with Levene´s test and if *p* < 0.05 the values were logarithmically transformed. Mixed models were used to test for interaction between catheter position and order of experiment period. Normally distributed data are presented as mean ± standard deviation (SD); data with a non-normal distribution are presented as median and interquartile range (IQR). The level of significance was set at *P* < 0.05.

## Results

All 18 piglets were included in the experiment and all of them completed the protocol. There were no major differences in vital parameters before and after the experiments between the groups (see Table 1). Outflow and inflow pressures in the dialysis circuit were within the pre-set normal range throughout the experiment for all animals. The time between periods 1 and 2 ranged between 5 and 30 min depending on calibration of ventilation and MAP goals.

**Table 1:** Vital parameters

Vital parameters	Before LPS	Before CRRT EJV	Before CRRT FV	After CRRT EJV	After CRRT FV
	Mean ± SD median(IQR)	Mean ± SD median(IQR)	Mean ± SD median(IQR)	Mean ± SD median(IQR)	Mean ± SD median(IQR)
Heart rate (bpm)	82 ± 16	148 ± 32	149 ± 31	162 ± 46	163 ± 32
MAP (mmHg)	80 ± 9.6	76 ± 6	77 ± 6	75 ± 9	79 ± 7
CO (ml/min)	2.4 ± 0.7	3.0 ± 0.9	2.8 ± 0.75	3.0 ± 1	2.8 ± 0.8
PAWP (mmHg)	21.5 ± 2.7	32 ± 14	28 ± 11	29 ± 13	34 ± 13
SpO_2_ (%)	99 (96–100)	99 (97–100)	98 (96–100)	100 (98–100)	96 (93–100)
Temp (°C)	36.8 ± 0.4	37.0 ± 1	37.0 ± 0.8	37.1 ± 0.7	37.1 ± 0.9
etCO_2_ (kPa)	4.7 ± 0.4	4.8 ± 0.8	5.2 ± 0.5	5.0 ± 0.5	5.1 ± 0.6
pH	7.47 ± 0.04	7.36 ± 0.05	7.36 ± 0.04	7.36 ± 0.05	7.37 ± 0.03
pCO_2_ (kPa)	5.0 ± 0.3	5.9 ± 0.6	6.0 ± 0.5	5.9 ± 0.5	5.8 ± 0.4
pO_2_ (kPa)	21 ± 3.5	16.0 ± 7.1	15.3 ± 6.9	13.9 ± 4.1	14.8 ± 6.3
HCO^−^_3_ (mmol/L)		22.7 ± 4.9	24.1 ± 1.5	23.9 ± 1.4	23.7 ± 1.5
ScvO_2_ (%)	48 ± 7	54 ± 13	55 ± 12	51 ± 15	55 ± 16
P-Hb (g/l)	78 ± 5	94 ± 9	97 ± 8	97 ± 10	96 ± 10
P-Potassium (mmol/L)	3.8 ± 0.2	3.9 ± 0.2	3.8 ± 0.2	3.9 ± 0.2	3.9 ± 0.2
P-Sodium (mmol/L)	136 ± 1.5	135 ± 1.3	135 ± 1.2	135 ± 1.3	135 ± 1.3
P-Calcium (mmol/L)	1.3 ± 0.1	1.4 ± 0.07	1.4 ± 0.07	1.4 ± 0.08	1.4 ± 0.07
P-Chloride (mmol/L)	104 ± 1.6	104 ± 1.2	104 ± 1.4	105 ± 1.5	105 ± 1.4
P-Glucose (mmol/L)	7.7 ± 1.2	7.8 ± 2.1	7.9 ± 2.1	7.3 ± 1.7	7.3 ± 1.7
P-Lactate (mmol/L)	1.6 ± 0.5	2.4 ± 1.2	2.4 ± 1.1	2.4 ± 1.3	2.5 ± 1.1

Vital parameters before LPS-infusion, before after CRRT (continuous renal replacement therapy) in the EJV (external jugular vein) or FV (femoral vein), bpm (beats per minute), MAP (mean arterial pressure), CO (cardiac output), PAWP (pulmonary artery wedge pressure), SpO_2_ (oxygen saturation), etCO_2_ (end tidal carbon dioxide), pCO_2_ (arterial carbon dioxide pressure) pO_2_ (arterial oxygen pressure), HCO_3_^−^ (bicarbonate), PA-pO_2_ (pulmonary artery oxygen pressure) ScvO_2_ (central venous oxygen saturation), Hb (haemoglobin), P (plasma), p (pressure)

**Fig. 3:**
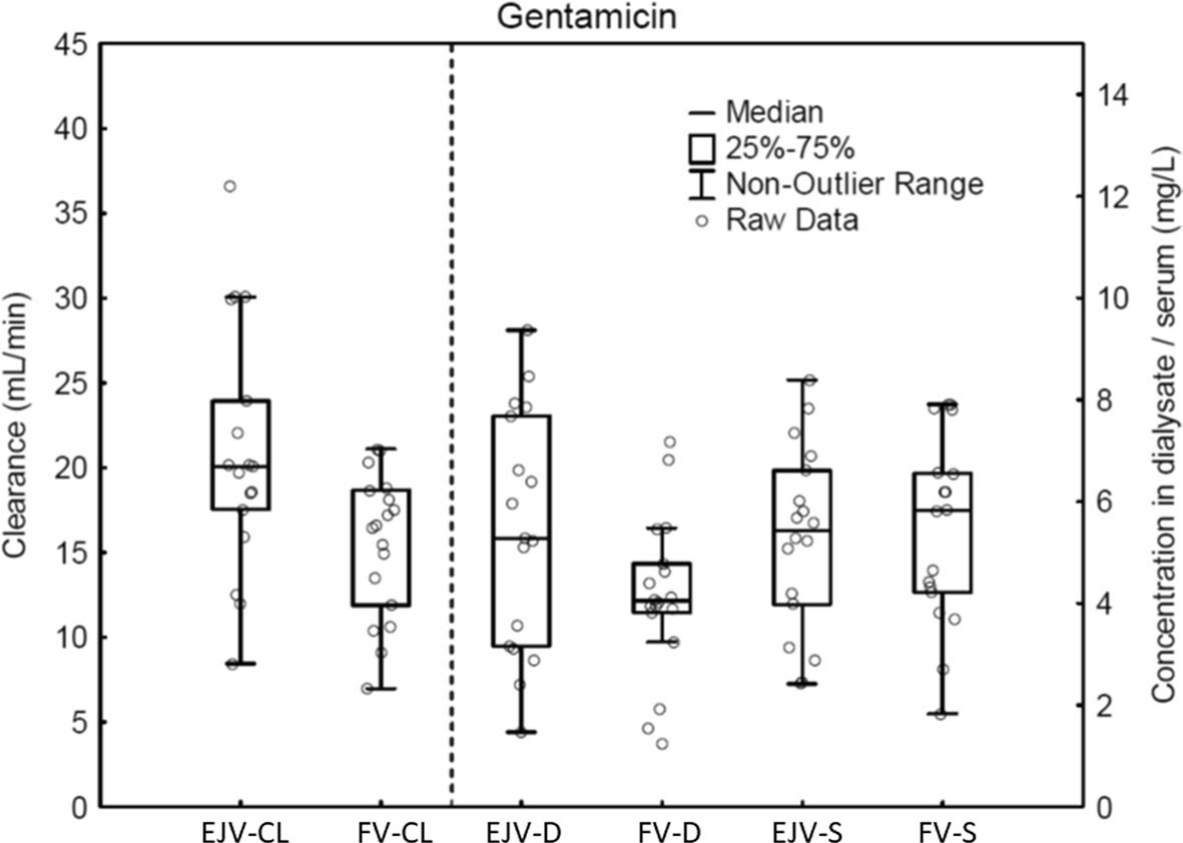
Clearance (CL), dialysate concentration (D) and serum concentration (S) of gentamicin (GM) and vancomycin (VM) with continuous renal replacement therapy in the external jugular vein (EJV) compared to the femoral vein (FV). Gentamicin infusion is in the EJV. When both the central venous dialysis catheter (CVDC) and gentamicin infusion are in the EJV, the clearance is higher than if the CVDC and gentamicin are in different vessels

The dialysate clearance of gentamicin was higher when CRRT was performed in a catheter placed in the EJV, i.e. close to the drug infusion, compared to in the FV (21.0 ± 7.3 vs 15.5 ± 4.2 mL/min (*p* 0.006)). The dialysate concentration of gentamicin was higher when CRRT was performed in a catheter placed in the EJV, compared to in the FV (5.4 ± 2.4 vs 4.1 ± 1.6 mg/L (*p* 0.013)). There was no difference in serum concentration of gentamicin between the EJV and FV (5.3 ± 1.8 vs 5.5 ± 1.9 mg/L (*p* 0.34)) (Fig. 3).

**Fig. 4:**
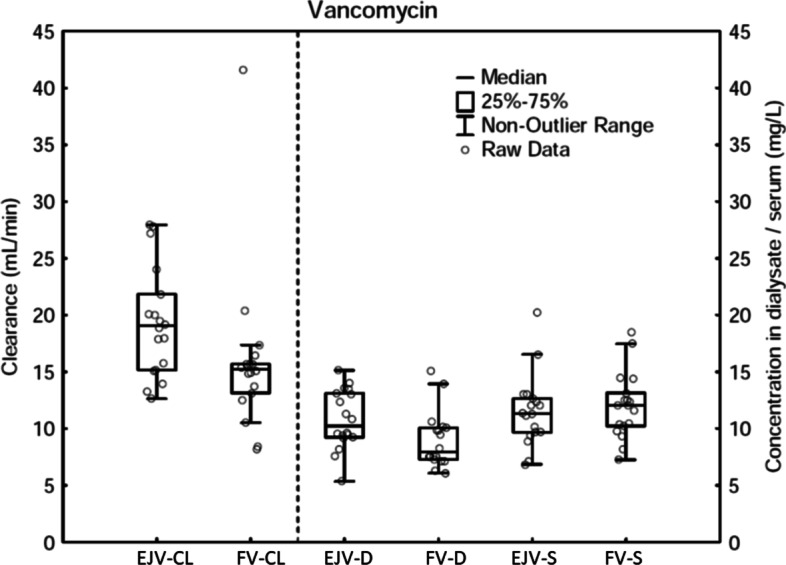
Clearance (CL), dialysate concentration (D) and serum concentration (S) of gentamicin (GM) and vancomycin (VM) with continuous renal replacement therapy in the external jugular vein (EJV) compared to the femoral vein (FV). Vancomycin infusion is in the EJV. When both the central venous dialysis catheter (CVDC) and vancomycin infusion are in the EJV, the clearance is higher compared to when the CVDC and vancomycin are in different vessels

Affirmative results were seen measuring vancomycin. Clearance (19.3 ± 4.9 vs 15.8 ± 7.1 mL/min (*p* 0.021)) and dialysate concentration (10.8 ± 2.6 vs 9.0 ± 2.5 mg/L (*p* 0.018)) were higher when CRRT was performed in a catheter placed in the EJV, i.e. close to the infusion rather than in the FV. The serum concentration of vancomycin showed no difference between the EJV and FV (11.3 ± 3.2 vs 12.1 ± 2.9 mg/L (*p* 0.089) (Fig. 4).

The circulatory response to LPS and the amount of noradrenaline required to reach a target MAP of 75 mmHg varied between the individuals. One piglet suffered a 2-min cardiac arrest but regained circulation after 1 dose of adrenaline and 2 min of cardiopulmonary resuscitation (CPR). In 15/36 experiment periods, the noradrenaline requirements were less than 1 µg/kg: 8/18 with CRRT in the EJV and 7/18 with CRRT in the FV. This can also be expressed as 1 µg/kg/ 30 min or 2 µg/kg/ 60 min, giving a dose as low as 0.03 µg/kg/min.

**Fig. 5:**
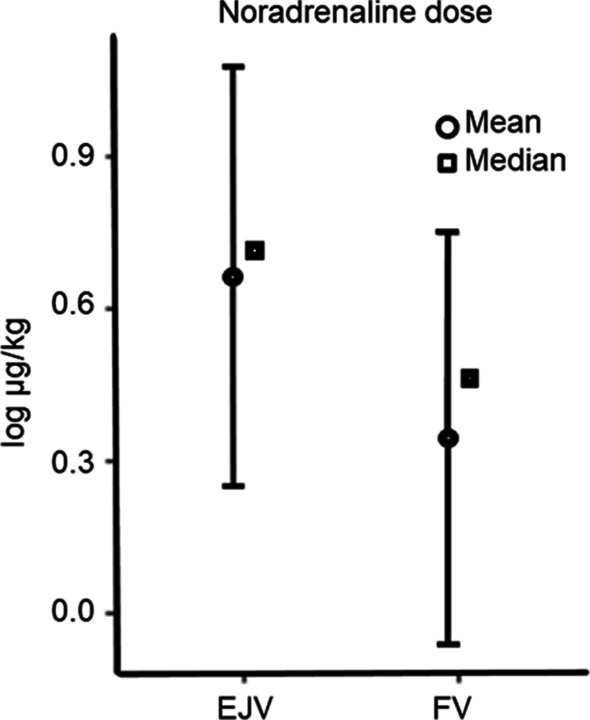
Noradrenaline dose needed to reach a target mean arterial pressure (MAP) of 75 mmHg, with continuous renal replacement therapy (CRRT) through a central venous dialysis catheter (CVDC) in the external jugular vein (EJV) or the femoral vein (FV). Noradrenalin infusion was in the EJV. The dose needed to reach the target MAP was higher when infusion was close to the CVDC, in the EJV, compared to when it was in separate vessels, in the FV

The variance of noradrenaline dose was higher when infusion was in the EJV, close to the CVDC. The dose of noradrenaline required to reach a MAP of 75 mmHg was higher when infusion and CRRT were in the EJV compared with when CRRT was in the FV (*p* = 0.02, Fig. 5).

The order of the experiment period did not affect clearance, dialysate or serum concentration of antibiotics. Position had a higher impact on dose than order. However, the noradrenaline doses were affected by order, resulting in a higher dose in experiment period 2.

## Discussion

The main finding in this study was that dialysate clearance of antibiotics was higher during CRRT when both catheter tips were in the EJV, close together, compared to when the catheters were in different vessels. This is analogous with the results from our previous study [8] and with the bench model from Kam [7].

Secondly, the noradrenaline dose to maintain a target MAP had a higher variance when the catheters were in the EJV, which could indicate that close placement of the catheter tips could lead to a less reliable drug concentration, due to direct aspiration.

The serum variation of drug concentrations, including antibiotics, is high in critically ill patients [17–19]. The reason is multifactorial and depending on factors like kidney function, body size and level of illness [18, 19]. This study indicates that placement of CVCs and CVDC could also be a factor that influences drug clearance, by direct aspiration. This phenomenon resembles recirculation, mostly studied in extra corporeal membrane oxygenation (ECMO) and in haemofiltration for chronic kidney failure. In ECMO, recirculation leads to a reduction of oxygenated blood and in renal replacement therapy it leads to a reduction of clearance. Direct aspiration of a drug will lead to higher clearance, a greater variance in drug requirement and possibly lower serum concentration [9–12].

In this study, there was no difference in antibiotic serum concentration, which could indicate that under septic conditions the 30-min experiment period was insufficient to show a difference, or that the time between the periods was too short (Individual serum concentrations are presented in Additional file 1). Our previous study detected a difference in antibiotic serum concentration, but that model did not include LPS and endotoxaemic response. Even though LPS no longer is the most recommended model for human sepsis, the main reason for using LPS in this experimental model was to make the endotoxaemic response more reproducible, and thereby reduce the number of animals needed [20]. The time of 30 min in the experimental model was chosen so that the piglets would be approximately in the same septic phase in both the period with CRRT in EJV and FV. If however, higher dialysate clearance would be for hours or days, which often is the case clinically, there would be a risk of low serum concentration.

The physician’s choice as to where to place different central venous catheters is directed by guidelines [21]. There are no recommendations on how multiple catheters should be placed. In ECMO, there is more recirculation when the catheter for withdrawal of blood is close to the catheter for infusion of oxygenated blood [11, 12]. To avoid close proximity of the tips, Butt et al. looked at the consequence of placing the CVC and CVDC in left and right internal jugular vein, respectively. There was no difference in distance between the tips if the catheters were placed in the same or different internal jugular veins [22]. Different central venous catheters and cannulas are also known to migrate due to repositioning of the patients, diaphragm movements and also by accidental retraction of the catheter [23–25]. Hence, radiographic control of tip placement would not guarantee avoidance of direct aspiration. However, this study suggests that if the catheter tips are very close together, adjustment of the catheter could be appropriate to reduce the risk of direct aspiration. Even separating the catheter tips to different vessels, for example to the EJV and FV, could be relevant. It is not known whether there is a specific distance between the catheters at which no direct aspiration occurs.

In both ECMO and haemofiltration, a higher blood pump flow leads to a higher level of recirculation and one could speculate that the same could be true for direct aspiration [11, 26]. A lower blood pump flow would then be preferred to reduce direct aspiration. Due to the fact that this was not the scope of this study, we did not change blood pump flow during the experiments.

The study’s limitations are all those associated with animal models. The purpose of introducing LPS was to make the model resemble sepsis in a clinical setting in the ICU. However, it also made the inter-individual differences greater and could possibly have clouded the results. The noradrenaline infusion dose was also affected by the order of the experiment period, where the second period required higher noradrenaline than the first. This is possibly explained by the experimental model with continuous LPS infusion leading to a slight deterioration over time, even though the vital parameters did not differ between the periods.

The above-mentioned limitations shed light on the difficulty of demonstrating this phenomenon in a clinical ICU setting. This study shows a difference in clearance of antibiotics and in drug requirement. However, the clinical importance is unclear and depends on the action of the infused drug. There is growing evidence to advocate antimicrobial therapeutic drug monitoring: the results of this study would further support that regime and encourage monitoring of other drug concentrations, when possible [27]. The consequences of direct aspiration depend on the drug being given and its action, but could also lead to altering clinical decisions. Commonly used drugs in the ICU are not monitored by blood concentration but by clinical response. If direct aspiration, for example, necessitates a higher noradrenaline dose, it could, by extension, lead to the introduction of a second line of vasopressor, i.e. vasopressin, with potentially harmful effects.

## Conclusion

During CRRT, in an endotoxaemic model, dialysate clearance of antibiotics was higher if CVC and CVDC were placed in the same vessel and in the same setting the variance of noradrenaline dose, to maintain a predefined MAP, was also higher. The results in this study indicate that close placement of central venous catheter tips could lead to unreliable drug concentration, due to direct aspiration, during CRRT**.**

## Supplementary Information

**Additional file 1**. Individual serum concentrations of antibiotics, measured at 4 different time points. Before experiment period with CRRT in EJV or FV, and after the experiment periods.

## Data Availability

The dataset analysed during this study is available from the corresponding author on reasonable request.

## Abbreviations

AB: Antibiotic
Bpm: Beat per minute
C: Clearance
CPR: Cardiac pulmonary resuscitation
CO: Cardiac output
CVC: Central venous catheter
CVDC: Central venous dialysis catheter
D: Dialysate
CRRT: Continuous renal replacement therapy
ECMO: Extra corporeal membrane oxygenation
EJV: External jugular vein
etCO_2_: End tidal carbon dioxide
pCO_2_: Arterial carbon dioxide pressure
FV: Femoral vein
GM: Gentamicin
HCO_3_^−^: Bicarbonate
Hb: Haemoglobin
ICU: Intensive care unite
IQR: Inter quartile range
LPS: Lipopolysaccharide
MAP: Mean arterial pressure
NA: Noradrenaline
p: Pressure
P: Plasma
PA-pO_2_: Pulmonary artery oxygen pressure
PAWP: Pulmonary artery wedge pressure
pO_2_: Arterial oxygen pressure
S: Serum
SD: Standard deviation
SpO_2_: Oxygen saturation
ScvO_2_: Central venous oxygen saturation
VM: Vancomycin
