# Supplementary material for: In an endotoxaemic model, antibiotic clearance can be affected by different central venous catheter positions, during renal replacement therapy
Source: Intensive Care Med Exp. 2023 Jun 9;11:32. doi: 10.1186/s40635-023-00516-4 (PMC10250278; doi:10.1186/s40635-023-00516-4)
Supplement: Supplementary file 1 — Additional file 1. Individual serum concentrations of antibiotics, measured at 4 different time points. Before experiment period with CRRT in EJV or FV, and after the experiment periods. [file 40635_2023_516_MOESM1_ESM.docx]

|  | **Gentamicin serum concentration**  **(mg/L)** | | | |  | **Vancomycin serum concentration**  **(mg/L)** | | | |
| --- | --- | --- | --- | --- | --- | --- | --- | --- | --- |
|  | T1 | T2 | T3 | T4 |  | T1 | T2 | T3 | T4 |
| Animal |  |  |  |  |  |  |  |  |  |
| 1 | 1.6 | 4.4 | 4.5 | 5.1 |  | 9 | 9.8 | 6.9 | 11.3 |
| 2 | 2.7 | 3.1 | 2.9 | 4.3 |  | 14.5 | 13.0 | 10.1 | 12.1 |
| 3 | 4.1 | 4.2 | 4.2 | 4.2 |  | 5.4 | 7.2 | 5.5 | 6.8 |
| 4 | 4.0 | 4.0 | 4.0 | 4.7 |  | 12.1 | 11.1 | 12.8 | 10.4 |
| 5 | 7.1 | 7.4 | 7.4 | 7.8 |  | 6.6 | 13.0 | 13.0 | 14.5 |
| 6 | 5.5 | 5.8 | 5.8 | 5.3 |  | 9.6 | 9.3 | 9.3 | 8.9 |
| 7 | 5.7 | 5.7 | 5.7 | 6.6 |  | 6.1 | 7.1 | 7.1 | 8.2 |
| 8 | 3.6 | 3.8 | 3.8 | 6.0 |  | 9.1 | 10.2 | 10.2 | 9.4 |
| 9 | 1.7 | 1.8 | 2.0 | 2.4 |  | 12.9 | 12.5 | 10.1 | 12.1 |
| 10 | 2.9 | 2.5 | 2.5 | 2.7 |  | 9.6 | 11.3 | 10.4 | 13.2 |
| 11 | 6.2 | 5.8 | 6.5 | 6.2 |  | 10.4 | 12.6 | 11.9 | 12.5 |
| 12 | 6.2 | 6.2 | 6.3 | 6.6 |  | 11.8 | 12.1 | 9.6 | 10.2 |
| 13 | 6.8 | 5.2 | 6.2 | 5.8 |  | 11.7 | 9.7 | 10.2 | 10.4 |
| 14 | 7.6 | 7.9 | 7.8 | 7.8 |  | 13.1 | 12.3 | 11.6 | 12.4 |
| 15 | 9.9 | 7.9 | 8.5 | 8.4 |  | 15.6 | 18.5 | 17.4 | 20.2 |
| 16 | 6.4 | 6.9 | 7.1 | 7.8 |  | 13.4 | 16.6 | 14.9 | 17.4 |
| 17 | 6.9 | 6.6 | 4.7 | 5.6 |  | 17.6 | 14.4 | 12.4 | 12.0 |
| 18 | 2.6 | 2.9 | 3.5 | 3.7 |  | 10.9 | 9.7 | 9.8 | 11.6 |

*Additional file 1. Individual serum concentrations of antibiotics, measured at 4 different time points. T1 and T3 is before experiment period with CRRT in EJV or FV. T2 and T4 are after the experiment periods. Between T2 and T3 the CRRT site was shifted.*
